# Temporal recalibration for improving prognostic model development and risk predictions in settings where survival is improving over time

**DOI:** 10.1093/ije/dyaa030

**Published:** 2020-04-03

**Authors:** Sarah Booth, Richard D Riley, Joie Ensor, Paul C Lambert, Mark J Rutherford

**Affiliations:** 1 Biostatistics Research Group, Department of Health Sciences, University of Leicester, Leicester, UK; 2 Centre for Prognosis Research, Research Institute for Primary Care and Health Sciences, Keele University, Keele, UK; 3 Department of Medical Epidemiology and Biostatistics, Karolinska Institutet, Stockholm, Sweden

**Keywords:** Prognostic models, temporal recalibration, period analysis, up-to-date survival predictions, flexible parametric survival models, Cox proportional hazards models

## Abstract

**Background:**

Prognostic models are typically developed in studies covering long time periods. However, if more recent years have seen improvements in survival, then using the full dataset may lead to out-of-date survival predictions. Period analysis addresses this by developing the model in a subset of the data from a recent time window, but results in a reduction of sample size.

**Methods:**

We propose a new approach, called temporal recalibration, to combine the advantages of period analysis and full cohort analysis. This approach develops a model in the entire dataset and then recalibrates the baseline survival using a period analysis sample. The approaches are demonstrated utilizing a prognostic model in colon cancer built using both Cox proportional hazards and flexible parametric survival models with data from 1996–2005 from the Surveillance, Epidemiology, and End Results (SEER) Program database. Comparison of model predictions with observed survival estimates were made for new patients subsequently diagnosed in 2006 and followed-up until 2015.

**Results:**

Period analysis and temporal recalibration provided more up-to-date survival predictions that more closely matched observed survival in subsequent data than the standard full cohort models. In addition, temporal recalibration provided more precise estimates of predictor effects.

**Conclusion:**

Prognostic models are typically developed using a full cohort analysis that can result in out-of-date long-term survival estimates when survival has improved in recent years. Temporal recalibration is a simple method to address this, which can be used when developing and updating prognostic models to ensure survival predictions are more closely calibrated with the observed survival of individuals diagnosed subsequently.

**Key Messages**

If survival has been improving over time, standard full cohort models can under-estimate survival.
Period analysis uses a more recent subset of data to produce survival estimates which are more up-to-date, however it reduces the sample size and number of events used in the analysis.
Temporal recalibration combines the sample size advantages associated with full cohort analysis with the up-to-date estimates produced with period analysis.
Temporal recalibration can be used at the model development stage or to update existing prognostic models when new data becomes available.

## Introduction

For individuals diagnosed with a particular disease or health condition, prognostic models can provide outcome predictions and aid treatment decisions.^1^^,^^2^ In this article, we focus on the outcome of time-until-death from colon cancer and survival predictions, however the approach can be generalized. Prognostic models contain multiple predictors and are typically developed using a regression format such as logistic, Cox or a parametric survival model. It is often of interest to provide survival predictions at different time points, such as 1, 5 and 10 years after diagnosis. For 10-year predictions, it is necessary to have a model development dataset that includes individuals who were diagnosed at least 10 years ago, such that the analysis has sufficient follow-up length. However, this can lead to out-of-date (miscalibrated) survival predictions for recently diagnosed individuals if there have been improvements in survival over calendar time: e.g. in recent years treatment may have improved survival compared with 5 or 10 years earlier. Improvements in survival for colorectal cancer have been reported in a number of different countries.^3–6^

With the development of online tools and apps, survival estimates from prognostic models have become more accessible. Some models such as PREDICT, a prognostic model for breast cancer,^7^ and QCancer, a prognostic model for colorectal cancer,^8^ are freely available online for both clinicians and the public. The survival estimates produced from these, and many other webtools, are from a standard full cohort analysis approach. Such models may produce survival predictions that under-estimate the true survival probability of recently diagnosed patients (and conversely over-estimate the actual risk of adverse outcomes).

Period analysis has been used in population-based cancer studies to obtain up-to-date estimates of survival^9–12^ and in this article we explore its use in the development and updating of prognostic models. Period analysis defines a recent time window and only the risk-time and events that fall within this window contribute to the estimates of the hazard rates and predictor effects.^13^ This method is not commonly used for prognostic models, however Keogh *et al*.^14^ produced survival predictions for cystic fibrosis patients using period analysis. A disadvantage with period analysis is that it results in a reduction of sample size for model development. This could be particularly problematic in small datasets, when there are rare predictor patterns or rare events, and may lead to a low number of events per predictor parameter, which increases the potential for model overfitting.^15^

In this article we introduce a new approach, called temporal recalibration, that combines the use of full cohort analysis, period analysis and recalibration methods. Specifically it aims to maximize the use of data toward model development, with the full dataset used to model predictor effects and the baseline survival recalibrated in a recent time window to produce more up-to-date survival predictions for new individuals. We illustrate and compare these methods using an example of colon cancer from the Surveillance, Epidemiology, and End Results (SEER) Program database.^16^

## Methods

### Cox proportional hazards models and post-estimation of the baseline

Cox proportional hazards (PH) models are frequently used to develop prognostic models.^17^ The model is of the form:

$$h(t;x_{i})=h_{0}(t)e^{{\beta x}_{i}\;}$$

with $h(t;x_{i})$ the hazard function, $h_{0}(t)$ the baseline hazard function and $\beta x_{i}$ the prognostic index.^18^

The cumulative hazard function $H(t;x_{i})$ must be approximated to calculate survival predictions as it is not directly modelled. This can be achieved post-estimation using a non-parametric approach, or by a smoother using fractional polynomials or splines.^7^^,^^19^ In this article, restricted cubic splines are used to create a smooth approximation of the log cumulative baseline hazard post-estimation. The same knot locations as the flexible parametric survival models (FPMs) (see Supplementary File 1, available as Supplementary data at *IJE* online) were used to ensure a fair comparison. The baseline survival curve was approximated by ${\hat{S}}_{0}\left(t\right)=e^{-\hat{H_{0}}(t)}$, and survival predictions for individuals with different values of the prognostic index by $\hat{S}\left(t;x_{i}\right)={\hat{S}}_{0}{\left(t\right)}^{e^{{\hat{\beta }x}_{i}}}$.

It is possible to extend these models to include time-dependent predictor effects (i.e. non-proportional hazards). Period analysis^20^ (see the Period analysis section) can be performed using delayed entry techniques.

### Flexible parametric survival models

Although Cox models are widely used for prognostic modelling, FPMs have several advantages. FPMs directly model the log baseline cumulative hazard function which allows for smooth survival curves to be produced during model development, without the need for post-estimation smoothing.^21^ It remains straightforward to include time-dependent predictor effects^22^ and incorporate delayed entry. FPMs use restricted cubic splines to directly model the baseline $\ln\left[H_{0}\left(t;x_{i}\right)\right]$ (see Supplementary File 1, available as Supplementary data at *IJE* online). A prognostic model can be written in the following form where $\zeta \left(\ln\left(t\right)|\gamma ,k_{0}\right)$ is the restricted cubic spline function and $\beta x_{i}$ is the prognostic index.^23^
 

$$\ln\left[H(t;x_{i})\right]=\zeta \left(\ln\left(t\right)|\gamma ,k_{0}\right)+\beta x_{i}$$

### Period analysis

**Figure 1:**
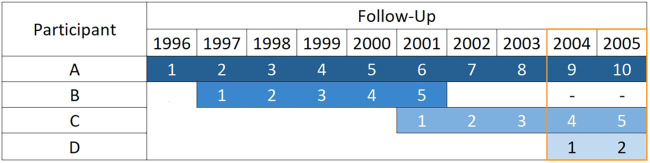
Contribution of follow-up time from four hypothetical participants (diagnosed 1 January) to a 2-year period window of 2004–05.

Period analysis, in the context of population-based cancer data, was developed by Brenner and Gefeller.^20^ Only individuals who contribute follow-up time during the period window are included in the analysis to estimate predictor effects and baseline survival (see Table 1). This reduces the sample size since people who experienced the event before the window (e.g. Participant B, see Figure 1) are excluded. Only the events that occur within the window are considered in the analysis and therefore the choice of window width is a balance between ensuring up-to-date survival estimates and having sufficient events (and events per predictor parameter). The width of the window could be determined by meeting the criteria defined by Riley *et al*.^15^ Further details and a sensitivity analysis of using different window widths are included in Supplementary File 4, available as Supplementary data at *IJE* online.

**Table 1.** Summary of the data used for the estimation of the baseline and predictor effects for each method

Method	Baseline	Predictor effects
Full cohort	Full	Full
Temporal recalibration	Recent	Full
Period analysis	Recent	Recent

Delayed entry techniques are used to left truncate the follow-up time of people diagnosed before the window so that the short-term hazard rates are only estimated from those diagnosed within or shortly before the period window (e.g. Participant D, see Figure 1).

This method has been shown to produce more up-to-date survival estimates than full cohort analysis in population-based cancer settings for many types of cancer in different countries^9–12^ and is used routinely within international cancer survival comparisons.^3^^,^^24^

### Temporal recalibration

A key disadvantage with period analysis is the reduction in sample size and number of events for model estimation. To address this, we propose temporal recalibration, which combines the sample size advantages associated with the full cohort analysis with the up-to-date predictions from period analysis.

The process of fitting a temporal recalibration model is as follows. (i) Fit a survival model using the full cohort dataset to estimate the predictor effects using all individuals. (ii) Recalibrate the model by re-estimating the baseline using the subset of individuals from a period analysis sample, while holding the predictor effect estimates from step (i) fixed.

Recalibrating the baseline in a recent period analysis sample allows for improvements in survival to be captured and leads to more up-to-date predictions. Under proportional hazards the model can be written in the following form for FPMs:

$$\ln\left[H_{\mathrm{new}}(t;x_{i})\right]=\zeta_{\mathrm{new}}(\ln\left(t\right)|\gamma ,k_{0})+{\mathrm{offset}(\mathrm{PI}}_{i})$$

where $\zeta_{\mathrm{new}}\left(\ln\left(t\right)|\gamma ,k_{0}\right)$ is the updated spline function for the log cumulative baseline hazard function estimated in the recent period data, $k_{0}$ are the knot locations from the full cohort model and ${\mathrm{offset}(\mathrm{PI}}_{i})$ is the prognostic index estimated from the full cohort model as an offset term. Fixing the predictor effects with constraints when fitting in the period analysis sample would offer an equivalent approach.

For a Cox PH model it can be written as:

$$h_{\mathrm{new}}(t;x_{i})=h_{0_{\mathrm{new}}}(t)e^{\mathrm{offset}(PI_{i})\;}$$

where $h_{\mathrm{new}}(t;x_{i})$ and $h_{0_{\mathrm{new}}}\left(t\right)$ are the hazard and baseline hazard functions respectively, estimated on the recent time window, and ${\mathrm{offset}(\mathrm{PI}}_{i})$ is the prognostic index estimated from the full cohort model as an offset term.

As with period analysis, the choice of the window width is a bias-variance trade-off (see Supplementary File 4, available as Supplementary data at *IJE* online). The width of the window could possibly be reduced compared with a standard period analysis approach as it is only necessary to have a sufficient number of events to estimate the baseline (and not the predictor effects). In temporal recalibration we explicitly assume the predictor effects are the same as they were in the full cohort model (see Table 1).

### Assessing the performance of predictions

Marginal survival (i.e. average across all individuals) can be calculated both within-sample (i.e. in the same dataset used to develop the model) and out-of-sample (i.e. in new individuals) by calculating every individual’s predicted survival over time, and then averaging the survival curves:^25^
 

$$\bar{\hat{S}}\left(t\right)=\frac{1}{N}\sum_{i=1}^{n}\hat{S}\left(t;x_{i}\right)$$

Out-of-sample marginal survival predictions can be compared with the observed survival (Kaplan–Meier estimates) to determine the calibration of a model’s survival predictions for a new group of individuals.

Studying the marginal survival only assesses how well the model performs on average (sometimes referred to as calibration-in-the-large^1^^,^^26^), whereas calibration plots can be used to determine the model’s performance in different risk groups at particular time points. In this article the risk groups were defined by dividing the prognostic index from the full cohort models into 10 equally sized risk groups.

The *E*/*O* statistic quantifies calibration-in-the-large by comparing predicted or expected (*E*) outcome risk to the observed (*O*) risk through $\frac{E}{O}(t)=\frac{1-S_{\;\text{exp}\;}(t)}{1-S_{\mathrm{obs}}(t)}$. *E* is calculated from the marginal survival prediction from the model [S_exp_(*t*)] and *O* is from the observed Kaplan–Meier curve [S_obs_(*t*)]. A value of 1 indicates agreement^1^^,^^27^

Harrell’s c-index can be used to assess the concordance of survival predictions from proportional hazards models. A value of 1 indicates perfect concordance.^28^

We now compare full cohort, temporal recalibration and period analysis approaches using an illustrative example of colon cancer.

## Example

### Data

We used the public-access SEER database from the USA.^16^ The SEER program covers ∼34% of the US population and collects population-based data on all reported cases of cancer within the cancer registries included in the SEER program.^29^ The analysis was restricted to adults who were aged 18–99 years at the time of their diagnosis of colon cancer (ICD10 codes C18.0–C18.9). If there were any duplicates of the patient ID, only the first record was retained. Patients with an unknown survival time (recorded to the nearest month) or incomplete dates for their diagnosis or death were also excluded. Data from 1996–2015 were available for this analysis. As the aim was to identify which model gave better long-term survival predictions in new data, the data were split at 2005 for illustration purposes. Data from 1996–2005 were used to develop the models and a 2 year period window from 1 January 2004 to 31 December 2005 was used to fit the temporal recalibration and period analysis models. The data from 2006–15 were then used to validate the models. Baseline characteristics for the development dataset can be found in Table 2.

**Table 2.** Baseline characteristics of the 48 861 participants in the development dataset once participants with missing predictor values were removed. Mean (SD) is presented for continuous variables and *n* (%) for categorical variables

Variable	Mean (SD) or *n* (%)
Age	70.1 (13.0)
Sex	
Male	23 674 (48.5%)
Female	25 187 (51.5%)
Race	
White	42 296 (86.6%)
Black	6565 (13.4%)
Stage at diagnosis	
Stage 1	18 469 (37.8%)
Stage 2	21 529 (44.1%)
Stage 3	8863 (18.1%)
Grade of tumour at diagnosis	
Grade 1	5496 (11.2%)
Grade 2	32 992 (67.5%)
Grade 3	9871 (20.2%)
Grade 4	502 (1.0%)

### Models

Cause-specific Cox and FPMs were fitted, meaning that deaths due to causes other than colon cancer were censored. Age at diagnosis, stage at diagnosis (localized, regional, distant), grade of the tumour (I–IV), sex and race (restricted to White and Black patients only) were included as predictors. Age was modelled using restricted cubic splines with three degrees of freedom, and stage, grade, sex and race were modelled categorically. All predictors were forced to be included (i.e. there was no variable selection). For the FPMs, five degrees of freedom were used to model the log baseline cumulative hazard and, to simplify the process of recalibration, the baseline splines were not orthogonalized. Example code used to fit these models is provided in Supplementary File 2, available as Supplementary data at *IJE* online. In this illustrative example, any participants with missing predictor values were excluded in order to more easily compare the approaches, though in practice multiple imputation is usually preferable.

Age at diagnosis was winsorized^30^ to provide more stability in the extremes by adding an additional constraint forcing the splines to be constant for the top and bottom 2% of the age distribution.^31^ In further analyses, the PH assumption was relaxed using time-dependent predictor effects for age and stage. To compare the model predictions from these three approaches, the marginal predicted survival for the 5601 patients diagnosed in 2006 was calculated using each model and compared with the observed Kaplan–Meier estimates. This was further assessed through calibration plots at 10 years after diagnosis.

All analyses were performed using Stata Version 15.0.^32^ FPMs were fitted using the user-written package stpm2^33^ and Harrell’s c-index was calculated for these models using the user-written package stcstat2.^34^

## Results

In terms of predictor effect estimates, the log hazard ratios and standard errors were very similar regardless of whether Cox models or FPMs were used (Table 3). The log hazard ratios were fairly similar for full cohort and period analysis, however the standard errors from the period analysis approaches were around twice as large due to the reduction in sample size. Overfitting was minimial due to the large number of events relative to the number of predictor parameters, highlighted by a uniform shrinkage factor^35^ for the full cohort model of 0.999.

**Table 3.** Comparison of the sample size, number of events, log hazard ratios (HR) and standard errors (s.e.) of the log hazard ratios for the categorical predictors in each model

		Flexible parametric survival model		Cox proportional hazards model	
		Full cohort	Period analysis	Full cohort	Period analysis
Sample size		48 861	33 197	48 861	33 197
Number of events		12 040	2900	12 040	2900
Predictor effects: log HR (s.e. of log HR)	Female	−0.05 (0.018)	−0.10 (0.038)	−0.05 (0.018)	−0.10 (0.038)
	Black	0.24 (0.025)	0.27 (0.051)	0.24 (0.025)	0.27 (0.051)
	Stage 2	1.15 (0.031)	1.18 (0.060)	1.15 (0.031)	1.18 (0.060)
	Stage 3	2.98 (0.031)	2.92 (0.062)	2.96 (0.031)	2.90 (0.062)
	Grade 2	0.22 (0.039)	0.09 (0.073)	0.22 (0.039)	0.10 (0.073)
	Grade 3	0.68 (0.041)	0.55 (0.078)	0.67 (0.041)	0.54 (0.078)
	Grade 4	0.81 (0.088)	0.78 (0.146)	0.79 (0.088)	0.75 (0.146)

**Figure 2:**
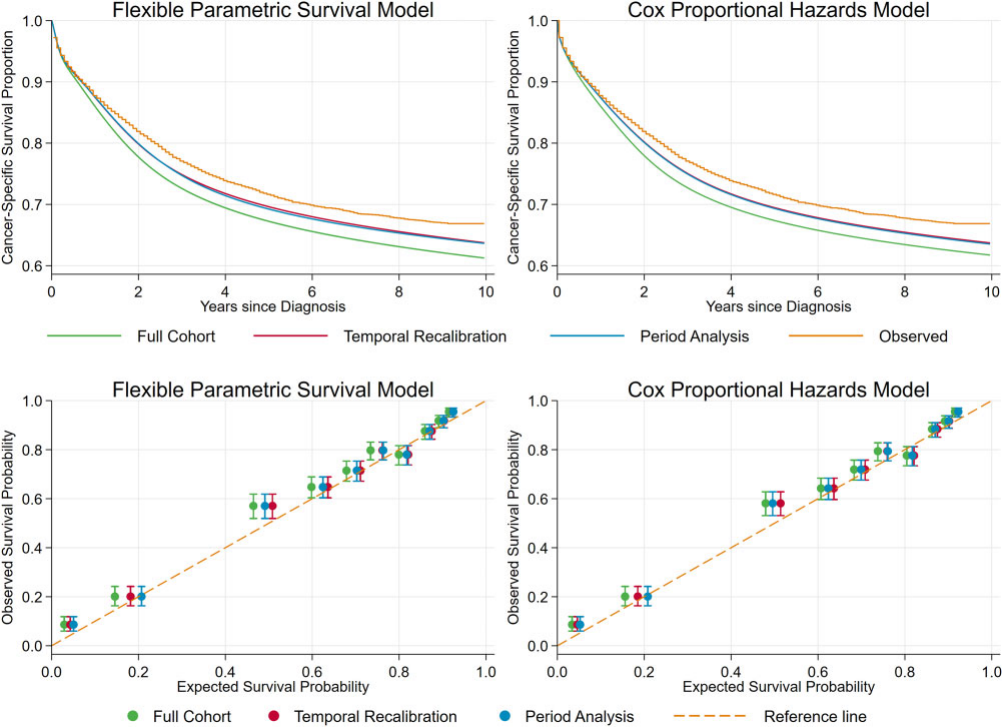
External validation of the models to assess the calibration of survival predictions for new patients (diagnosed in 2006 with follow-up data until 2015). Top: comparison of marginal observed (Kaplan–Meier) and predicted survival from each model. Note: The predictions from the temporal recalibration and period analysis models overlay almost exactly. Bottom: 10-year calibration plots comparing the observed and predicted cancer-specific survival probabilities from each model.

Similar marginal survival predictions were produced regardless of whether Cox or FPMs were used when predicting for patients diagnosed in 2006 (Figure 2). The marginal survival predictions for temporal recalibration and period analysis were very similar and consistently provided more well-calibrated estimates than the standard full cohort model. The survival probability is under-estimated for all risk groups in the full cohort analysis models, and in 9 of the 10 groups the predictions are the furthest from the reference line. However, using temporal recalibration, all the predicted survival estimates increase and agree more closely with the Kaplan–Meier estimates. Although the marginal survival predictions from the temporal recalibration and period analysis models are very similar, small differences in predicted survival can be seen for the highest risk groups. Including time-dependent effects for age and stage in the FPM improves the calibration in the third highest risk group, however there is very little difference in the marginal survival estimates, see Supplementary File 3, available as Supplementary data at *IJE* online.

A comparison of the model performance in terms of calibration and concordance of survival predictions is displayed in Table 4. Calibration improves by 0.02 by performing temporal recalibration which is large at the population level and improves the net benefit of the model.^36^ In other scenarios, the difference may be greater if there have been more substantial changes in baseline survival over calendar time. As the predictor effects for the temporal recalibration models are constrained to be the same as those from the full cohort model, Harrell’s c-index will always be the same for these models. In this example, the predictor effects for the period analysis models were also very similar and therefore Harrell’s c-index is the same to three decimal places.

**Table 4.** Comparison of model performance in the validation dataset. The difference in observed and predicted marginal survival at 10 years after diagnosis [*S*_obs_(10) – *S*_exp_(10)], the ratio of expected to observed risk at 10 years after diagnosis (*E*/*O*) and Harrell’s c-index

Model	*S*_obs_(10) – *S*_exp_(10)^a^	$\frac{E}{O}$(10)	Harrell’s c-index
Full cohort: FPM	0.056	1.169	0.788
Full cohort: Cox	0.051	1.155	0.788
Temporal recalibration: FPM	0.031	1.094	0.788
Temporal recalibration: Cox	0.031	1.095	0.788
Period analysis: FPM	0.032	1.098	0.788
Period analysis: Cox	0.033	1.101	0.788

a *S*_obs_(10), Kaplan–Meier estimate at 10 years after diagnosis; *S*_exp_(10), 10 year marginal survival prediction from the model.

### Updating prognostic models

Temporal recalibration can also be used to produce up-to-date survival estimates when new data become available by simply re-estimating the baseline without the need for repeating the model-building process or re-estimating the predictor effects. This is akin to previous work by Riley *et al*.,^37^ Schuetz *et al*.^38^ and Steyerberg^26^ that show how recalibrating the baseline hazard in new (local) settings can be important. To illustrate this, prognostic models were fitted using FPMs with data from 1986–95, and data from 1996–2005 was used to update these models. As stage was only available from 1995 onwards, only age, sex, race and grade were included as predictors, and for simplicity PHs was assumed. Table 5 defines the models M1-M6 that were compared in this analysis.

**Table 5.** Comparison of the data used to estimate the predictor effects and baseline of each flexible parametric survival model

Model	Description	Data for predictor effects	Data for baseline
M1	Original full cohort model	1986–95	1986–95
M2	Full cohort model with all available data	1986–2005	1986–2005
M3	Full cohort model with most recent data	1996–2005	1996–2005
M4	Temporal recalibration of M1	1986–95	Period window 2004–05
M5	Temporal recalibration of M3	1996–2005	Period window 2004–05
M6	Period analysis	Period window 2004–05	Period window 2004–05

**Figure 3:**
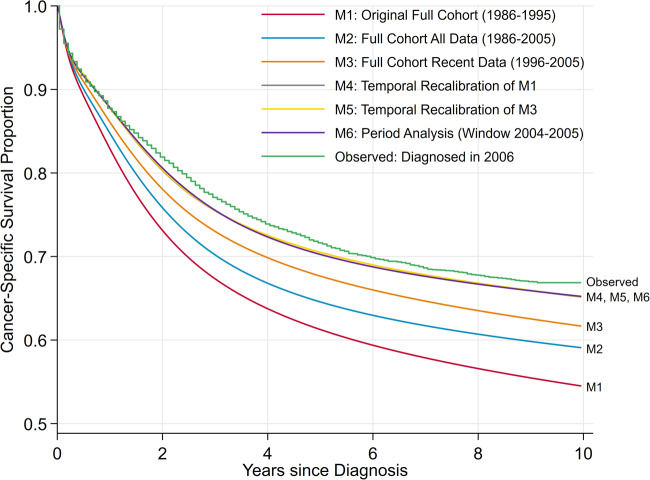
Comparison of marginal observed (Kaplan–Meier curve) and predicted survival from the original and updated models. Note: The predictions from the temporal recalibration and period analysis models overlay almost exactly.

To illustrate the difference in survival predictions for these models, 10-year survival was estimated for patients diagnosed in 2006 and compared with the Kaplan–Meier estimates for these patients, see Figure 3.

Using the original model (M1) resulted in a difference between the observed and predicted survival of 0.12, which was reduced to 0.08 by using a longer timespan (M2) and 0.05 by using a more recent cohort (M3). Temporal recalibration models (M4 and M5) and the period analysis model (M6) produced the closest estimates which differed by <0.02. Performing temporal recalibration improves the calibration of the full cohort models by at least 0.03 and a larger improvement of >0.10 is observed when recalibrating the original full cohort model. Despite different models being temporally recalibrated, the predictions overlaid exactly. This demonstrates that temporal recalibration is appropriate in this example since the predictor effects do not greatly change over time and therefore it is only necessary to re-estimate the baseline.

## Discussion

Often there are large underlying improvements in survival over the follow-up available in a model development dataset, which presents a challenge for subsequently making predictions for newly diagnosed patients. We have shown that survival predictions from prognostic models developed using a standard full cohort approach underestimate survival of recently diagnosed patients. However, more up-to-date, and thus accurate, survival predictions can be produced by developing prognostic models using temporal recalibration, where the baseline hazard is recalibrated in a subset of most recent data. This idea is similar to the approach of period analysis, but has the additional benefit of more precisely estimating predictor effects as it uses all the data to estimate the prognostic index.

Unlike period analysis, it is possible to directly apply temporal recalibration to a range of existing prognostic models (i.e. Cox PH models, FPMs with time-dependent effects) to update the survival predictions without the need of repeating the model-building process or re-estimating predictor effects. No additional data are required, only a period analysis sample of the most recent data is needed to re-estimate the baseline and produce more up-to-date predictions which better reflect the survival of those currently being diagnosed. We have also shown the importance of regularly updating prognostic models when new data become available and how this can easily be achieved using temporal recalibration.

We have used SEER public use data for colon cancer patients, with a range of predictors in order to illustrate the approach. For cancer sites and settings with smaller improvements over calendar time, the predicted survival estimates from a standard and temporally recalibrated approach would differ less. However, the approach would still be valid in this case. In this example we only showed complete case analysis, however, temporal recalibration could be performed on imputed datasets and the survival predictions from the models could be combined using Rubin’s rules.^39^^,^^40^ Example code for fitting these models is included in Supplementary File 2, available as Supplementary data at *IJE* online.

Temporal recalibration assumes that the predictor effects are the same in the recent data as in the full cohort and therefore do not change as a function of diagnosis date. This is in contrast to period analysis which updates both the baseline and the prognostic index. Therefore, the parameter estimates from the full cohort and period analysis models can be informally compared to verify that this assumption is plausible. Further, careful consideration should be given to the consistency of predictor’s values over time, but this is an issue generally and not specific to the approach we outline here.

Temporal recalibration is a similar concept to model updating,^41^ in which the calibration of predictions from a previously developed prognostic model are externally validated using new data obtained from a more recent time point. In that setting, if the model consistently under or over predicts survival, it is recalibrated; typically predictor effects are kept fixed (i.e. as originally estimated), but the baseline is updated. The difference with temporal recalibration is that the period analysis sample (used for the recalibration) is not a separate dataset and has already been included in the full cohort model to estimate predictor effects.

An alternative to temporal recalibration and period analysis would be to model the year of diagnosis directly and then predict survival using the most recent year included in the model. This approach would make developing and updating existing prognostic models more challenging as it would require the year of diagnosis to be modelled appropriately, which may include time-dependent effects and non-linear terms. This method would also rely more heavily on extrapolation of effects when producing long-term survival predictions for the most recent calendar year. However, with temporal recalibration the long-term hazards are estimated directly from those included in the period window. With both temporal recalibration and modelling the year of diagnosis it may be necessary to consider interactions between predictor effects and year of diagnosis.^42^

Many existing prognostic models use the standard full cohort approach. We have illustrated that using temporal recalibration could update these survival predictions and be a more accurate reflection of the prognosis of patients who are currently being diagnosed.

## Funding

This work was supported by Cancer Research UK [C41379/A27583].

## Conflict of interest

None declared.

## Supplementary Material

dyaa030_Supplementary_Data
