# Supplementary material for: Temporal recalibration for improving prognostic model development and risk predictions in settings where survival is improving over time
Source: Int J Epidemiol. 2020 Apr 3;49(4):1316–25. doi: 10.1093/ije/dyaa030 (PMC7750972; doi:10.1093/ije/dyaa030)
Supplement: dyaa030_Supplementary_Data [file dyaa030_supplementary_data.zip › dyaa030-suppl_data/ije-2019-05-0635-File011.pdf]

S.1 Flexible Parametric Survival Models (FPM)

The restricted cubic spline function is defined by a set of knot locations which split the time-scale into intervals. A greater number of knots allows for more complex shapes to be captured. The resulting function is a set of constrained cubic polynomials where the function and its 1st and 2nd derivatives are constrained to be 0 at the knots, ensuring a smooth function. Restricted cubic splines have additional constraints that the function is linear before the first knot and after the last knot[1].

Flexible parametric survival models are modelled on the log cumulative hazard scale, where $\zeta(\ln(t)\,|\boldsymbol{\gamma},\boldsymbol{k_0})$ is the restricted cubic spline function and $\boldsymbol{\beta x}_i$ is the prognostic index.

$$\ln[H(t;\boldsymbol{x}_i)] = \zeta(\ln(t)\,|\boldsymbol{\gamma},\boldsymbol{k_0}) + \boldsymbol{\beta x}_i$$

The restricted cubic spline function is constructed of K knots (vector $\boldsymbol{k_0}$), derived variables z_i and parameters γ_i[1]

$$\zeta(\ln(t)\,|\boldsymbol{\gamma},\boldsymbol{k_0}) = \gamma_0 + \gamma_1 z_1 + \cdots + \gamma_{K-1} z_{K-1}$$

$$z_1 = \ln(t)$$

$$z_j = (\ln(t) - k_j)_+^3 - \phi_j(\ln(t) - k_1)_+^3 - \left(1 - \phi_j\right)(\ln(t) - k_K)_+^3 \quad j = 2, \ldots K-1$$

$$\phi_j = \frac{k_K - k_j}{k_K - k_1}$$

References:

[1] Hinchliffe SR, Lambert PC. Flexible parametric modelling of cause-specific hazards to estimate cumulative incidence functions. *BMC Med Res Methodol* 2013; **13**.
