# Supplementary material for: Temporal recalibration for improving prognostic model development and risk predictions in settings where survival is improving over time
Source: Int J Epidemiol. 2020 Apr 3;49(4):1316–25. doi: 10.1093/ije/dyaa030 (PMC7750972; doi:10.1093/ije/dyaa030)
Supplement: dyaa030_Supplementary_Data [file dyaa030_supplementary_data.zip › dyaa030-suppl_data/ije-2019-05-0635-File012.pdf]

S.2 Example Stata Code

S.2.1 Program to perform temporal recalibration

```
capture program drop recalibrate
program define recalibrate, rclass
	syntax [varlist(default=none)], [model(string)]
	if "`model'"!="" {
		estimates restore `model'
	}

	* Main Effects
	local savedmodel=`"`e(cmdline)'"'
	gettoken modelcov options: savedmodel, parse(,)
	gettoken model cov: modelcov
	fvexpand `cov'

	foreach var in `r(varlist)' {
		local c = _b["`var'"]
		constraint free
		constraint `r(free)' _b["`var'"] = `c'
		local constraint_list "`constraint_list' `r(free)'"
	}

	* Time Dependent Effects (if any) - for flexible parametric survival models
	if "`e(cmd)'"=="stpm2" {
		foreach var in `e(tvc)' {
			forvalues i=1/`e(df_`var')' {
				local c = _b[_rcs_`var'`i']
				constraint free
				constraint `r(free)' _b[_rcs_`var'`i'] = `c'
				local constraint_list "`constraint_list' `r(free)'"
			}
		}
	}

	* Return Constraint List
	return local constraints = "`constraint_list'"

	* Knot Locations - for flexible parametric survival models
	if "`e(cmd)'"=="stpm2" {
		return local knots = e(bhknots)
		return local bknots = e(boundary_knots)
		foreach var in `e(tvc)' {
			return local tvcknots_`var' = e(tvcknots_`var')
		}
	}

end
```

S.2.2 Example code for flexible parametric survival models

Flexible parametric survival models can be fitted using the *stpm2* package[1] in Stata[2]. Marginal survival predictions can be made after fitting the models with the *predict* command using the *meansurv* option.

Full Cohort Model

Use *stset* to prepare the data for survival analysis, where dx = date of diagnosis, cause 21040 = death due to colon cancer, yydx = year of diagnosis. Only include those diagnosed in or after 1996 and restrict follow-up to the end of 2005. Fit a flexible parametric survival model (without orthogonalising the baseline splines) and store the model to be used for temporal recalibration.

```
stset exit if yydx>=1996, origin(dx) fail(cause==21040) scale(365.24) ///
exit(time mdy(12,31,2005))
```

```
stpm2 agercs* female black stage2 stage3 grade2 grade3 grade4, scale(hazard) ///
df(5) noorthog
```

```
estimates store cohort
```

Temporal Recalibration Model

Use *stset* with a period window of 2004-2005 (enter and exit option) to define the period analysis sample. Use the *recalibrate* command to obtain constraints and knot locations to re-fit the model. This updates the baseline survival but constrains the predictor effects to be the same as the full cohort model.

```
stset exit if yydx>=1996, origin(dx) fail(cause==21040) scale(365.24) ///
enter(time mdy(1,1,2004)) exit(time mdy(12,31,2005))
```

```
recalibrate, model(cohort)
```

```
stpm2 agercs* female black stage2 stage3 grade2 grade3 grade4, scale(hazard) ///
noorthog constraints("`r(constraints)'") bknots("`r(bknots)'") knots("`r(knots)'")
```

Note: Non-proportional hazards models can also be temporally recalibrated. For example, a full cohort model could be fitted to include time-dependent effects for stage with 3 degrees of freedom by using *tvc(stage2 stage3) dftvc(3)* when fitting the model.

This model can then be temporally recalibrated by additionally specifying the knot locations for the time dependent effects using *tvc(stage2 stage3) knotstvc(stage2 "`r(tvcknots_stage2)'" stage3 "`r(tvcknots_stage3)'")* .

Period Analysis Model

Use the same *stset* with a period window of 2004-2005 and fit a flexible parametric survival model.

*stset exit if yydx>=1996, origin(dx) fail(cause==21040) scale(365.24) ///
enter(time mdy(1,1,2004)) exit(time mdy(12,31,2005))*

stpm2 agercs female black stage2 stage3 grade2 grade3 grade4, scale(hazard) ///
df(5)*

S.2.3 Example code for Cox proportional hazard (PH) models

Cox PH models can be fitted using the *stcox* command[3] using the same *stset* commands as the flexible parametric survival models.

Full Cohort Model

Use *stset* to prepare the data and then fit a Cox PH model. Calculate the prognostic index in order to fit the temporal recalibration model.

*stset exit if yydx>=1996, origin(dx) fail(cause==21040) scale(365.24) ///
exit(time mdy(12,31,2005))*

stcox agercs female black stage2 stage3 grade2 grade3 grade4*

predict prognosticindex_cohort, xb

Temporal Recalibration Model

Use *stset* to define the period analysis sample and then fit a Cox PH model using the prognostic index from the full cohort as an offset term. This is equivalent to constraining each of the predictor effects to be the same. If the PH assumption is not satisfied and time-dependent predictor effects are included, it is not possible to use the prognostic index as an offset term as it becomes a function of time. However, it is possible to temporally recalibrate a non-PH flexible parametric survival model using the constraints with *stpm2*.

*stset exit if yydx>=1996, origin(dx) fail(cause==21040) scale(365.24) ///
enter(time mdy(1,1,2004)) exit(time mdy(12,31,2005))*

stcox, estimate offset(prognosticindex_cohort)

Period Analysis Model

Use *stset* to define the period analysis sample and then fit a Cox PH model.

*stset exit if yydx>=1996, origin(dx) fail(cause==21040) scale(365.24) ///
enter(time mdy(1,1,2004)) exit(time mdy(12,31,2005))*

stcox agercs female black stage2 stage3 grade2 grade3 grade4*

S.2.4 Multiple Imputation

If multiple imputation has been performed, Rubin's rules can be used to combine the model coefficients and produce survival predictions. The following code is for fitting flexible

parametric survival models, however a similar method can be applied to Cox proportional hazards models.

Full Cohort or Period Analysis:

mi stset is used to prepare the data and then *mi estimate* can be used to fit full cohort or period analysis models.

```
mi estimate, post cmdok saving(mi_model, replace): ///
stpm2 agercs* female black stage2 stage3 grade2 grade3 grade4, scale(hazard) df(5)
```

Temporal Recalibration

To fit temporal recalibration models to multiply imputed datasets, a full cohort model can be fitted in each of the imputed datasets and then within the imputed dataset it can be temporally recalibrated. Rubin's rules can then be applied to produce the final model coefficients and produce survival predictions. In the example code below, 10 imputed datasets were used.

```
forvalues i = 1/10 {
preserve
mi extract `i', clear

stset exit if yydx>=1996, origin(dx) fail(cause==21040) scale(365.24) ///
exit(time mdy(12,31,2005))
stpm2 agercs* female black stage2 stage3 grade2 grade3 grade4, scale(hazard) ///
df(5) noorthog

stset exit if yydx>=1996, origin(dx) fail(cause==21040) scale(365.24) ///
enter(time mdy(1,1,2004)) exit(time mdy(12,31,2005))
stpm2 agercs* female black stage2 stage3 grade2 grade3 grade4, scale(hazard) ///
noorthog constraints("`r(constraints)'") bknots("`r(bknots)'") knots("`r(knots)'")

restore
}
```

References:

[1] Lambert PC, Royston P. Further development of flexible parametric models for survival analysis. *Stata J* 2009; **9:**265–290.

[2] StataCorp. 2017, Stata Statistical Software: Release 15. College Station, TX: StataCorp LLC.

[3] StataCorp. 2017, Stata Survival Analysis, Reference Manual, Release 15. College Station, TX: Stata Press.
