# Supplementary material for: Temporal recalibration for improving prognostic model development and risk predictions in settings where survival is improving over time
Source: Int J Epidemiol. 2020 Apr 3;49(4):1316–25. doi: 10.1093/ije/dyaa030 (PMC7750972; doi:10.1093/ije/dyaa030)
Supplement: dyaa030_Supplementary_Data [file dyaa030_supplementary_data.zip › dyaa030-suppl_data/ije-2019-05-0635-File013.pdf]

S.3 Flexible parametric survival models with time-dependent effects

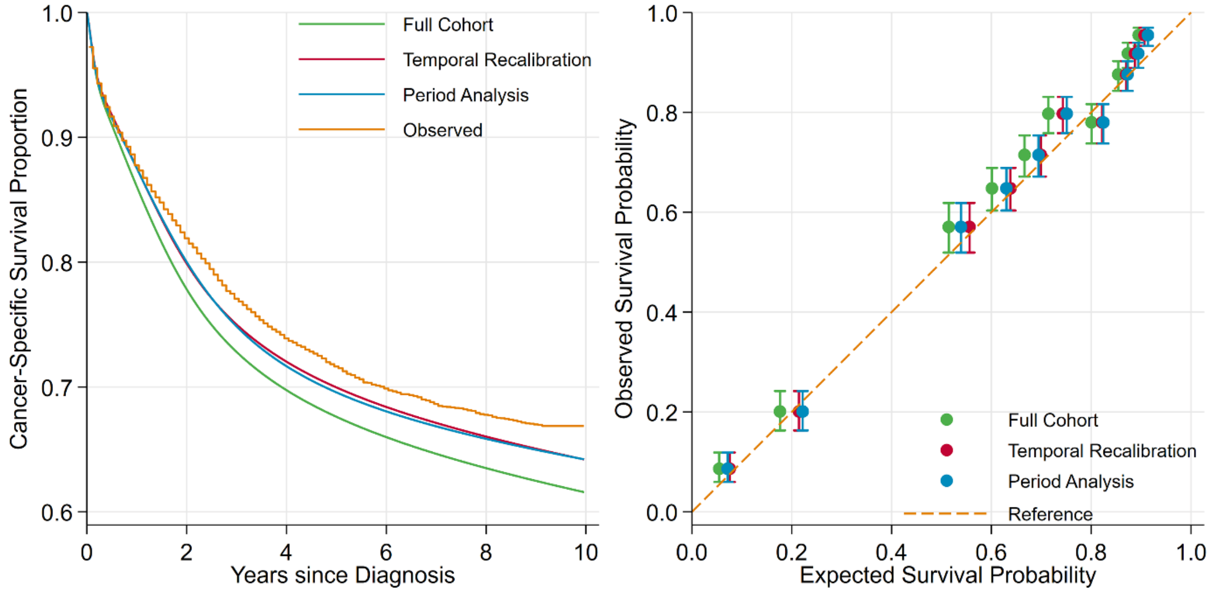

Figure 1: Assessing the calibration of predictions from flexible parametric survival models with time-dependent effects for age and stage. Left: Comparison of 10-year marginal survival predictions. Right: 10-year calibration plot comparing the observed and predicted cancer-specific survival probabilities.
