# Supplementary material for: Temporal recalibration for improving prognostic model development and risk predictions in settings where survival is improving over time
Source: Int J Epidemiol. 2020 Apr 3;49(4):1316–25. doi: 10.1093/ije/dyaa030 (PMC7750972; doi:10.1093/ije/dyaa030)
Supplement: dyaa030_Supplementary_Data [file dyaa030_supplementary_data.zip › dyaa030-suppl_data/ije-2019-05-0635-File014.pdf]

S.4 Sensitivity Analysis for Period Analysis Window Width

In the main analysis, a 2 year period window of 1st January 2004 – 31st December 2005 was used. The sensitivity of the results was assessed by fitting a series of period analysis and temporal recalibration proportional hazards models with varying window widths ranging from 4 years (1st January 2002 – 31st December 2005) to 3 months (1st October 2005 –31st December 2005).

Table 1: Comparison of sample size, number of events, number of events per predictor parameter (EPP) and the uniform shrinkage factor for the full cohort model and range of period analysis models

Type of Analysis	Sample Size	Number of Events	EPP	Uniform Shrinkage Factor
Full Cohort	48 861	12 040	1204	0.999
4 Year Window	38 395	5680	568	0.998
2 Year Window	33 197	2900	290	0.997
1 Year Window	30 427	1500	150	0.994
6 Month Window	28 896	774	77.4	0.989
3 Month Window	28 099	383	38.3	0.975

It can be seen in Table 1 that when the window width was reduced from 2 years to 3 months, 85% of participants were still included in the analysis but only 13% of the events were retained.

Riley et al. recommend including a sufficient number of events to ensure that the uniform shrinkage factor is at least 0.9[1]. Due to the relatively low number of predictors used in this model, all of these period analysis models would be suitable as the uniform shrinkage factor is at least 0.975.

Since the window widths are appropriate for period analysis models they are consequently suitable to use for temporal recalibration models since only the baseline is re-estimated in this subset of data. However, for period analysis models there must be a sufficient number of events to estimate both the baseline and the predictor effects.

Table 2 compares the log hazard ratio and its standard error for the female predictor parameter from a full cohort model and a range of period analysis models. Decreasing the window width from a 2 year window to a 3 month window results in a standard error almost 3 times greater. This is an advantage of temporal recalibration since the predictor effects are estimated on the full cohort dataset which maximises the use of the data and results in smaller standard errors.

Table 2: Comparison of the log hazard ratio (HR) and its standard error for the female predictor parameter from a full cohort model and period analysis models with varying window widths

Type of Analysis	log HR	Standard Error of log HR
Full Cohort	-0.047	0.018
4 Year Window	-0.105	0.027
2 Year Window	-0.103	0.038
1 Year Window	-0.132	0.053
6 Month Window	-0.074	0.073
3 Month Window	-0.062	0.104

Figure 1 compares the 10-year marginal survival predictions for the temporal recalibration and period analysis models with the different period analysis windows. In the temporal recalibration models it can be seen that the model with a 4 year window produces the lowest marginal survival predictions since this is closest to the full cohort model and includes the most out-of-date data. The marginal survival predictions from the other models agree very closely at 10 years after diagnosis, however there is some variation at around 5 years after diagnosis.

In the period analysis models there is greater variation in the predictions since the subset of recent data is also used to estimate the predictor effects and changes in these, in addition to changes in the baseline survival, affect the marginal survival curves. This is a further advantage of temporal recalibration since the survival predictions are less sensitive to the window width as only the baseline is re-estimated in the window.

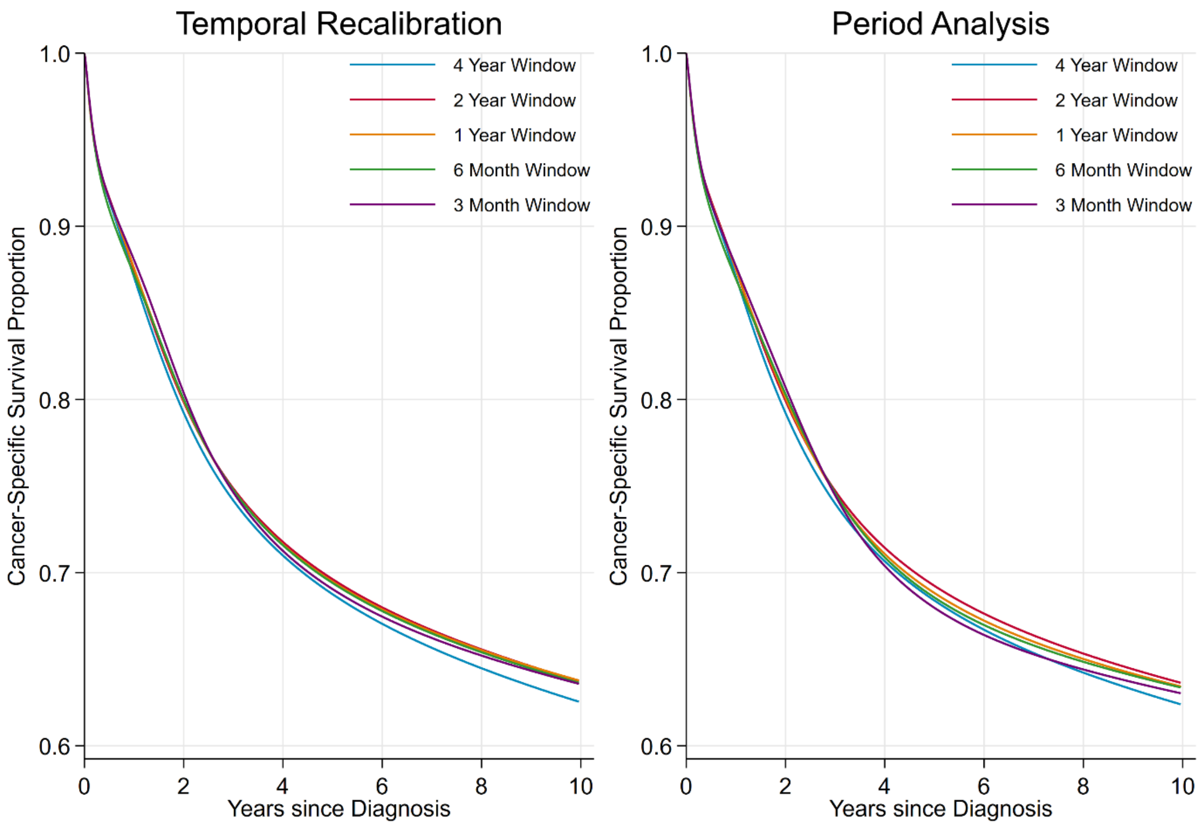

Figure 1: Comparison of marginal survival predictions for temporal recalibration and period analysis models fitted with varying window widths.

Figure 2 compares the 10-year calibration plot for the temporal recalibration and period analysis models for a 2 year period window, as used in the main analysis of the paper, against models with a 3 month period window. It can be seen that the marginal survival predictions in each risk group for the temporal recalibration models overlay exactly. However, there is more variation in the period analysis models as the predictor effects are different in each of the period analysis models. There would be greater variation in the calibration plots at around 7 years after diagnosis since there are larger differences in the marginal survival predictions (see Figure 1).

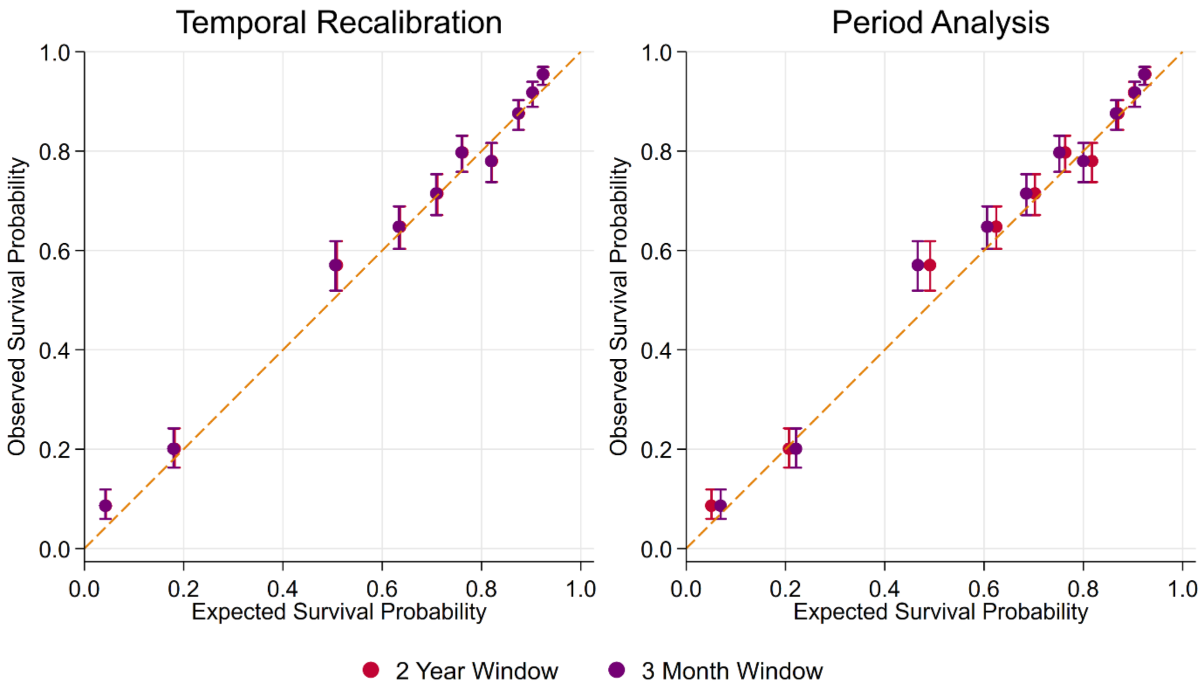

Figure 2: Comparison of 10-year calibration plots for temporal recalibration and period analysis models fitted using a 2 year window and a 3 month window.

In this example, there are a large number of events in the development dataset and therefore the width of the period analysis window does not greatly impact the marginal survival predictions. The choice of window width is a bias-variance trade-off as reducing the window width produces more up-to-date survival predictions but there is greater uncertainty in estimating the predictor effects and the baseline hazard. The choice of window width is highly dependent on the number of events in the development dataset and careful thought must be given to ensure that a sufficient number of events are used to develop the required prognostic model.

References:

[1] Riley RD, Snell KI, Ensor J et al. Minimum sample size for developing a multivariable prediction model: PART II - binary and time-to-event outcomes. *Stat Med* 2019; **38:**1276–1296.
